# Hydrogel-based neural engineering for skin wound healing

**DOI:** 10.3389/fcell.2026.1797417

**Published:** 2026-05-28

**Authors:** Yibo Zhang, Xilin Liu, Guangzhi Wu

**Affiliations:** 1 Department of Wound Repair, Plastic and Reconstructive Microsurgery, China-Japan Union Hospital of Jilin University, Changchun, China; 2 Department of Hand and Foot Surgery, China-Japan Union Hospital of Jilin University, Changchun, China

**Keywords:** exosomes, hydrogel-based materials, neuro-driven skin regeneration, neurotrophic factors, wound healing

## Abstract

Neuro-driven skin regeneration represents an emerging paradigm in wound healing that integrates peripheral nerve repair with functional skin restoration. Hydrogels serve as versatile platforms for supporting such dual tissue regeneration, owing to their biocompatibility, tunable physicochemical properties, and ability to mimic the extracellular matrix. Recent advances have enabled the development of multifunctional hydrogels that combine biophysical and biochemical cues—including conductive materials, bioactive molecules, and exosomes—to create healing microenvironments that promote nerve growth, angiogenesis, and tissue repair, particularly in challenging conditions such as diabetic ulcers and chronic wounds. This review examines the molecular mechanisms underlying neural regulation of wound healing, focusing on sensory neuron-derived factors, neuro-immune crosstalk, neurovascular integration, and the emerging role of neurogenic exosomes as central signaling hubs. Furthermore, design principles for hydrogel materials—including natural, synthetic, and composite systems—are explored alongside smart responsive hydrogels that adapt to dynamic wound environments, thereby enabling controlled therapeutic delivery. Current trends integrating wearable bioelectronics, artificial intelligence (AI), and bioengineered scaffolds are discussed in the context of real-time monitoring and personalized therapy. While neurogenic hydrogels show significant promise for clinical translation, their development requires rigorous preclinical validation and well-designed human trials. Beyond skin regeneration, these materials hold potential for nerve repair, bone healing, and cardiac tissue engineering, highlighting their versatility as therapeutic solutions across diverse physiological systems. Future efforts should focus on leveraging AI to optimize hydrogel formulations, advancing stem cell-based therapies, and establishing standardized metrics for treatment efficacy.

## 1 Introduction

**FIGURE 1:**
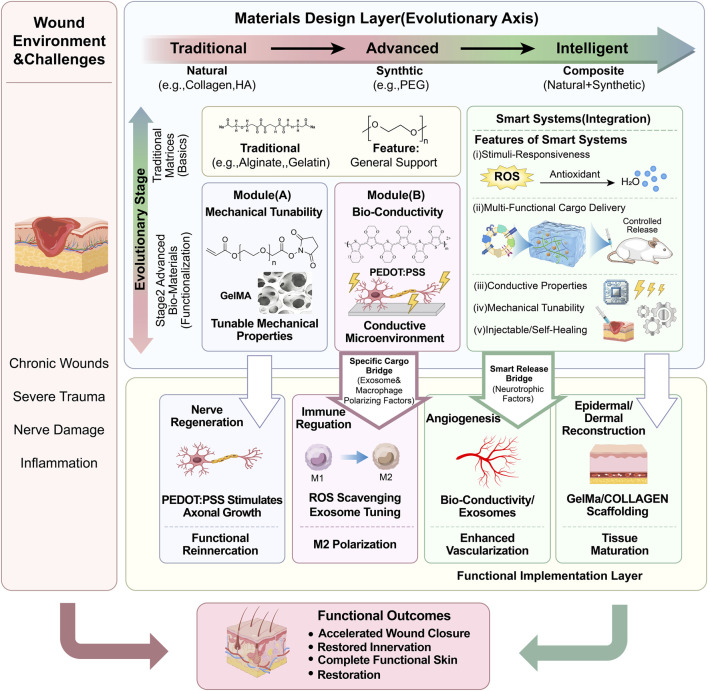
The multifunctional neurogenic hydrogel platform for functional skin reconstruction.

Hydrogel-based neuro-driven skin wound healing is a regenerative medicine approach that leverages peripheral nerve regeneration for the functional repair of difficult-to-heal skin wounds. Full restoration of structural integrity and sensory-motor function necessitates the synchronization of nerve regeneration with skin restoration, which is essential for treating severe trauma, burns, and chronic wounds. Owing to their biocompatibility, adjustable physicochemical properties, and extracellular matrix (ECM) mimicking capabilities, hydrogels serve as a versatile platform for dual tissue regeneration (Mokarram et al., 2017; Tan et al., 2023). Hydrogels, a class of soft biomaterials, can be broadly divided into natural, synthetic, and composite types. Natural hydrogels—such as gelatin, hyaluronic acid (HA), alginate, and chitosan—display inherent biocompatibility and bioactivity, alongside cell-adhesive motifs that support Schwann cell attachment, axonal extension, and neural stem cell (NSC) survival. These attributes are ideal for constructing nerve guidance conduits (NGCs) and neuro-permissive microenvironments. Synthetic hydrogels, including poly (ethylene glycol) (PEG) and poly (vinyl alcohol) (PVA), provide precise control over mechanical properties, crosslinking density, and degradation kinetics. These characteristics enable the development of reproducible scaffolds capable of delivering neurotrophic factors (NTFs) in a spatiotemporally controlled manner. Composite hydrogels integrate natural and synthetic components to achieve both biological recognition and tunable physical properties, allowing for the inclusion of conductive nanomaterials that mimic the electrophysiological niche of native neural tissue. In the context of neuro-driven skin wound healing, the structural and functional properties of hydrogels are increasingly engineered to target the neuro-immune-vascular axis, supporting nerve regeneration, modulating macrophage polarization, and enabling the sustained release of neurogenic exosomes and bioactive cues. Such an integrated design philosophy addresses the multifactorial challenges associated with chronic skin wounds, where impaired innervation represents a critical yet often overlooked pathological feature (Tibatan et al., 2025) (Figure 1). Furthermore, engineering hydrogels as smart responsive systems—sensitive to pH, reactive oxygen species (ROS), or enzymatic activity—enables their dynamic adaptation to the evolving wound microenvironment, a feature that is particularly advantageous when combined with neural regenerative strategies.

Recent advancements include the development of multifunctional hydrogels that establish a pro-healing environment through the integration of physical and chemical signals. For instance, chitosan hydrogels reinforced with reduced graphene oxide (rGO) exhibit improved mechanical properties, electrical conductivity, and biodegradability, which support skin tissue development and direct neurite outgrowth (Hartwell et al., 2015). The addition of rGO provides the scaffold with enhanced structural integrity and enables the electrical signal transmission required for neural differentiation and functional recovery (Mahheidari et al., 2024). Additionally, these hydrogels allow for the sequential delivery of therapeutic agents, such as salvianolic acid A, CXCL12, and plant-derived exosomes, which promote neuronal development, mitigate cellular senescence, and recruit mesenchymal stem cells (MSCs) (El Soury et al., 2023). This programmed release system achieves optimal efficacy by synchronizing the delivery of substances with the natural kinetics of wound healing and nerve regeneration (Gao et al., 2024).

Another significant advancement involves the application of hydrogel-based NGCs for the treatment of peripheral nerve abnormalities. Although autologous nerve grafts remain the clinical standard, their utility is limited by donor site morbidity and restricted tissue availability. Hydrogel-filled NGCs, such as those composed of fibrin-collagen matrices, have shown potential in promoting axonal regeneration and myelination, particularly in critical-sized nerve gaps (Kuna et al., 2022). Functionalized self-assembling peptides and aligned fibrin fibers have been demonstrated to enhance nerve fiber density and myelination in these conduits, almost achieving autograft performance levels (Yang et al., 2022). These aligned structures provide directional guidance to regenerating axons, creating an environment that is conducive to nerve repair, while bioactive peptides promote Schwann cell maturation and neurite development (Mazio et al., 2024).

The coupling of angiogenesis and neurogenesis represents another vital element of hydrogel-based wound healing strategies. Vascularization is essential for supplying oxygen and nutrients to regenerating tissue, while nerve regeneration is necessary for restoring sensory and motor function. Hydrogels designed as “all-in-one” smart dressings (ASD) integrate angiogenic and neuro-regenerative cues on a single substrate, enabling simultaneous microvascularization and neural repair (Hu et al., 2023). For example, systems utilizing zeolite-imidazolate frameworks (ZIF-67) anchored with vanadium oxide (VO_2​_) induce angiogenesis through the on-demand release of cobalt ions (Co^2+^) in response to the acidic wound microenvironment (Na et al., 2022). Furthermore, the continuous release of ligustroflavone, ginsenoside Rg1, and CXCL12 promotes MSC recruitment and initiates neural differentiation, thus achieving *in situ* nerve regeneration (Choi et al., 2021). Beyond accelerating wound closure, this integrated approach supports the restoration of functional skin appendages, including sweat glands and hair follicles (Yan et al., 2024).

The application of pre-vascularized dermal replacements (PVD) further demonstrates the potential of hydrogel-based skin regeneration approaches. These constructs have been shown to accelerate vascularization, innervation, and re-epithelialization in full-thickness skin defects. PVDs are composed of fibroblasts embedded within a fibroblast-secreted ECM and a capillary-like network (Lee et al., 2020). This biomimetic ECM facilitates graft integration with host tissue and encourages the development of functional skin appendages (Dong et al., 2021). Importantly, the early innervation observed in these grafts—occurring as early as 2 weeks post-implantation—highlights the importance of a vascularized, matrix-rich environment in promoting neural regeneration (Wang et al., 2023a).

The incorporation of bioactive nanoparticles, such as diopside (DNPs) and botulinum toxin A (BTX-A), into hydrogel matrices has been shown to enhance tissue repair and mitigate scarring (Hou et al., 2024). While DNPs act as pro-angiogenic agents that promote cell proliferation and reduce inflammation, BTX-A relieves muscular tension and facilitates wound healing (Li et al., 2023). The combination of these agents within a single hydrogel scaffold accelerates the healing process and improves the functional and aesthetic outcomes of the regenerated skin (Shan and Wu, 2024).

Hydrogel-based neuro-driven strategies represent a significant paradigm shift in regenerative medicine, providing a comprehensive approach to address the complex interplay between skin restoration and nerve regeneration. By leveraging the unique attributes of hydrogels—including biocompatibility, tunability, and the capacity for controlled bioactive release—these methods offer promising solutions for the treatment of severe trauma, burns, and chronic ulcers. To improve the therapeutic application of hydrogel scaffolds, future research should focus on optimizing the integration of biophysical and biochemical cues within these scaffolds and exploring advanced fabrication techniques. Continued developments in hydrogel-based therapies hold the potential to achieve true functional skin restoration and significantly improve the quality of life for patients with debilitating wounds.

## 2 Mechanisms of neural modulation in wound healing

Neural modulation in wound healing is mediated by a complex network linking the nervous system, immune responses, vascular remodeling, and tissue regeneration. Neural input coordinates wound repair by regulating blood flow, neurogenic inflammation, and the activity of specialized cells such as fibroblasts, endothelial cells, and macrophages (Alapure et al., 2018; Beauchene et al., 2023). Consistent with this view, denervation has been shown to impair granulation tissue formation, re-epithelialization, wound closure, angiogenesis, and connective tissue regeneration, highlighting the importance of neural signaling in maintaining the structural and functional integrity of the wound microenvironment (Alapure et al., 2018; Beauchene et al., 2023).

Beyond these general effects, neural regulation of wound healing involves distinct interconnected mechanisms. Sensory neuron-derived factors directly influence epithelial, vascular, and immune cells, while neural signals shape the bidirectional crosstalk between nerves and immune cells, thereby influencing inflammatory resolution and tissue repair. At the tissue level, neurovascular interactions provide trophic and metabolic support for regeneration, while neurogenic exosomes act as a signaling medium linking neural, immune, and vascular responses. The integration of these processes ultimately drives the structural reconstruction of the epidermis and dermis. Accordingly, this section examines these mechanisms, beginning with direct molecular regulation through neuro-immune and neurovascular interactions to exosome-mediated signaling and tissue reconstruction. Understanding these neural regulatory mechanisms provides the biological rationale for designing hydrogel-based platforms that can actively modulate the neuro-immune-vascular axis.

### 2.1 Direct regulation by sensory neuron-derived factors

**FIGURE 2:**
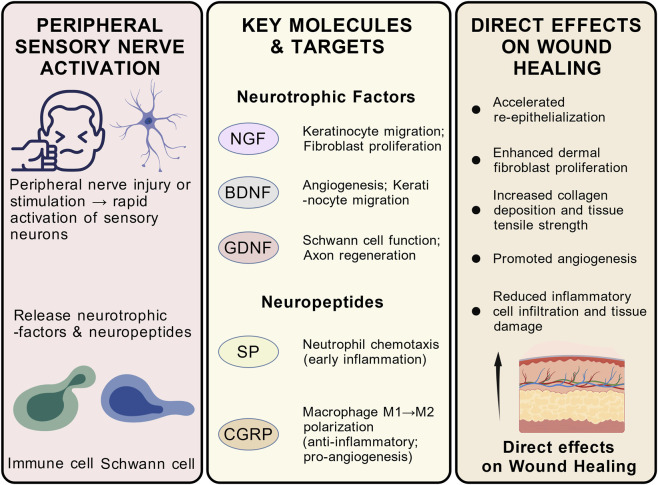
Neurogenic signaling and the immune - vascular regulatory network (Created with BioGDP.com).

Following injury, peripheral sensory neurons release a variety of signaling molecules, including substance P (SP), calcitonin gene-related peptide (CGRP), glial cell line-derived neurotrophic factor (GDNF), brain-derived neurotrophic factor (BDNF), and nerve growth factor (NGF). These factors constitute a signaling axis that directly regulates skin repair by acting on fibroblasts, keratinocytes, immune cells, and vascular endothelial cells (Figure 2).

NGF, the first NTF identified, is widely expressed in sensory neurons and non-neuronal cells, and can accelerate wound re-epithelialization by promoting keratinocyte migration and dermal fibroblast proliferation (Liu et al., 2021). Similarly, BDNF contributes directly to wound repair by regulating angiogenesis and keratinocyte migration and has been shown to improve tissue perfusion and regeneration in diabetic models (Ashrafi et al., 2016). GDNF acts as an important regulator of Schwann cell activity and axonal regeneration, and its sustained release from hydrogels can enhance reinnervation and support wound healing (Bi et al., 2025a).

In addition to NTFs, sensory neuropeptides exert direct effects on the early wound microenvironment. SP promotes neutrophil recruitment and participates in the initial inflammatory response. Meanwhile, CGRP exhibits pro-angiogenic and anti-inflammatory properties, and also promotes macrophage polarization toward the M2 phenotype. In denervated mice, the loss of CGRP markedly reduces wound re-epithelialization and immunomodulation, highlighting its importance in tissue repair (Lu et al., 2024). Furthermore, local CGRP administration in diabetic wounds was reported to accelerate wound closure while enhancing M2 polarization and angiogenesis (Li X. et al., 2025).

### 2.2 Bidirectional neuro-immune crosstalk

Neuro-immune crosstalk is a critical component of wound healing, in which neural signals shape immune responses and immune cells, in turn, participate in regenerative signaling. Sensory nerve terminals directly regulate the intensity and duration of inflammation through neuropeptides such as SP and CGRP. The former SP promotes neutrophil chemotaxis and supports the initial inflammatory response, while the latter drives macrophage polarization from the pro-inflammatory M1 phenotype toward the pro-reparative M2 phenotype (Lu et al., 2024).

The CSF1-BMP2 axis further illustrates this bidirectional interaction. Neurogenic colony-stimulating factor 1 (CSF1) induces the expression of bone morphogenetic protein 2 (BMP2) in macrophages, thereby activating downstream regenerative signaling and establishing a positive feedback loop between neural activity and immune-mediated repair (Wang S. et al., 2024). In diabetic wounds, which exhibit persistent inflammation and oxidative stress, therapeutic interventions can reshape neuro-immune coupling. Conductive hydrogels that initially promote Schwann cell migration and axonal regeneration have been reported to downregulate the expression of TNF-α and IL-1β, upregulate that of CD206, and restore macrophage homeostasis (Bi et al., 2025b). Similarly, the on-demand release of hydrogen sulfide (H_2_S) was shown to mitigate oxidative stress and inflammation while protecting mitochondrial activity in nerve cells, further linking immune resolution to neural repair (Dong et al., 2023).

Collectively, these findings indicate that neuro-immune crosstalk is not limited to inflammatory control but also helps determine the regenerative capacity of the wound microenvironment. Neurogenic exosomes may represent an additional level of immune modulation; however, because they connect neural, immune, and vascular responses more broadly (Fan et al., 2022), they are discussed separately in Section 2.4.

### 2.3 The neurovascular niche and its integration with the immune system

The neurovascular niche is a key regulatory unit in wound healing, in which nerves and blood vessels cooperate in the coordination of tissue repair. Sensory nerve-derived signals not only influence immune responses but also directly shape vascular function. In this context, the neuropeptide-mediated immune-vascular connection has emerged as an important component of regeneration. Sustained CGRP signaling improves endothelial function and promotes macrophage polarization toward the M2 phenotype through the inhibition of p53 signaling, thereby linking immune regulation with vascular remodeling during wound repair (Li X. et al., 2025).

Neurons and blood vessels often function as a symbiotic unit during skin regeneration. While sensory neurons directly promote angiogenesis through vascular endothelial growth factor (VEGF), newly formed blood vessels provide trophic support, oxygen, and metabolic sustenance for regenerating nerves. In addition, neurogenic guidance signals, such as semaphorin-3A, coordinate axonal guidance and vascular chemotaxis, forming a coupled microenvironment that supports both neural and vascular remodeling (Shi et al., 2022).

Immune regulation is integrated into this neurovascular niche rather than acting as a parallel process. Immunological remodeling can indirectly improve angiogenesis and tissue perfusion by promoting endothelial lumen formation and interacting with Schwann cell-mediated regenerative changes. In this setting, exosomes released from neurons and Schwann cells can function as signaling vehicles within the neuro-immune-vascular axis. Through the delivery of protein complexes and miRNAs, such as miR-124, miR-21, and miR-146, these vesicles promote macrophage reprogramming toward the M2 phenotype without the requirement for direct cell-to-cell contact (Ghosh and Pearse, 2023; Yin and Shen, 2024).

**FIGURE 3:**
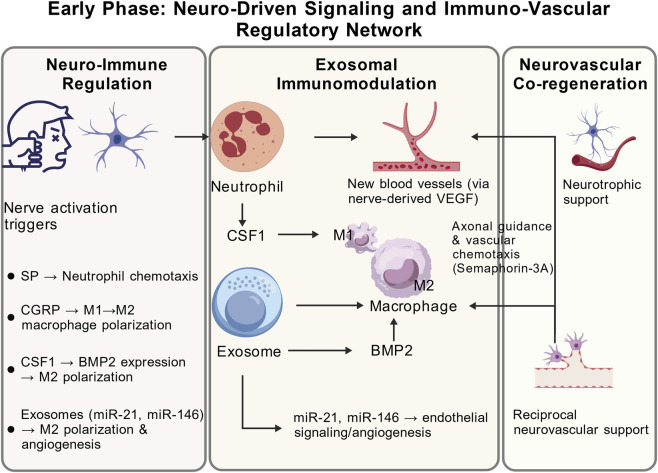
Key signaling molecules of peripheral sensory nerve activation and their direct effects on wound healing (Created with BioGDP.com).

From a biomaterial perspective, anchoring exosomes to hydrogels to achieve on-demand release may further optimize the temporal coupling between axonal regeneration and vascular maturation, while also spatially restricting peripheral diffusion and aligning delivery with the phases of inflammation, proliferation, and remodeling (Huang et al., 2023; Fan M.-H. et al., 2024) (Figure 3). However, because neurogenic exosomes function more broadly as a central signaling hub, their roles are discussed in greater detail in Section 2.4.

### 2.4 Neurogenic exosomes as a central signaling hub

Neurogenic exosomes constitute a vital signaling hub, integrating intercellular communication, regenerative regulation, and therapeutic delivery within a single acellular platform. These extracellular vesicles, typically 30–150 nm in diameter, are secreted by various neural and glial cell types—including astrocytes, microglia, and NSCs—and transport bioactive cargos such as lipids, proteins, and nucleic acids (Lai et al., 2022; Colvett et al., 2023). Their regulatory significance stems from their ability to transfer functional molecular information through which they influence neurogenesis, synaptic plasticity, and neuroinflammation (Yuan et al., 2023; Tang et al., 2022).

A primary function of neurogenic exosomes involved the regulation of neural regeneration through cargo-dependent signaling. NSC-derived exosomes can stimulate neuronal and glial proliferation by delivering miRNAs, such as miR-9, which targets Hes1, a transcriptional repressor of NSC differentiation (Ma et al., 2019). This neuroregenerative potential is influenced by cellular origin; for example, research indicates that exosomes derived from astrocyte-reprogrammed NSCs exhibit distinct neurogenic activity (Wang et al., 2021). In addition, neurogenic exosomes modulate oxidative stress and neuroinflammation. In spinal cord injury models, exosomes derived from human umbilical cord MSCs have been shown to promote neurite outgrowth and attenuate the detrimental effects of neurotoxic astrocytes, partially through miR-146a-5p-mediated regulation of the Traf6/Irak1/NF-κB pathway (Kumar et al., 2019; Joshi and Zuhorn, 2021). Similarly, exosomal miR-199a-3p and miR-145-5p promote neurite regeneration and functional recovery through the NGF/TrkA signaling pathway (Ortega-Pineda et al., 2022).

A second critical feature of neurogenic exosomes relates to their delivery capacity. NSC-derived exosomes interact with heparan sulfate proteoglycans on brain endothelial cells and subsequently undergo transcytosis across the blood-brain barrier, suggesting that they may serve as effective endogenous carriers for therapeutic proteins, miRNAs, and neuroprotective factors (Zou et al., 2022; Zhou et al., 2023). Their ability to transport transcription factors such as Ascl1, Brn2, and Myt1l further supports their potential to serve as nanoplatforms for gene- or oligonucleotide-based neural repair.

In the context of wound healing and tissue regeneration, neurogenic exosomes function as a key link among the neural, immune, and vascular dimensions of repair. Functional exosomes released by neurons and Schwann cells are enriched in miRNAs and NTFs. Notably, cortical neuron-derived exosomes rich in miR-124 can be gradually released from hydrogels to promote neural regeneration in injured tissues (Wang K. et al., 2024). Compared with direct cell transplantation, exosome-mediated acellular therapy offers a more manageable strategy for neuro-modulated wound healing (Fan et al., 2024b). Importantly, exosomes derived from neurons or Schwann cells and enriched in molecules such as miR-124, miR-21, and miR-146 drive macrophage M2 polarization, enhance endothelial angiogenic potential, and direct stem-cell differentiation. Thus, these vesicles function as a central signaling node within the neuro-immune-vascular axis, optimizing the wound microenvironment for functional skin regeneration (Fan et al., 2022).

### 2.5 Nerves in the structural reconstruction of skin

Nerves contribute directly to the structural reconstruction of skin by regulating both epidermal renewal and dermal remodeling. In the epidermis, resident stem cells are controlled by niche microenvironments that involve neural connections, while cutaneous nerves influence the fate and activity of Lgr6-marked epidermal stem cells during wound re-epithelialization (Huang et al., 2021). Denervation or the ablation of Lgr6 stem cells delays wound healing. Moreover, intravital imaging and single-cell lineage tracing experiments have shown that the loss of neural input promotes the differentiation of Lgr6+ cells, underscoring the importance of neural signals in maintaining epidermal stem-cell plasticity during repair (Huang et al., 2021).

In the dermis, peripheral nerves regulate ECM remodeling and contribute to the maintenance of its structural integrity through interactions with fibroblasts and other stromal cells (Broussard et al., 2021). Neural signaling pathways influence fibroblast activity and myofibroblast differentiation, thereby contributing to the transition from collagen III-dominant regeneration to a collagen I-dominant repair process during wound healing (Broussard et al., 2021). Nociceptive and sympathetic nerves also participate in the control of blood flow, immune responses, and matrix deposition, indicating that neural input is integrated into dermal remodeling rather than being limited to sensory transmission (Erbacher et al., 2024).

Advanced experimental approaches have provided further support for this structural role of nerves. Intravital imaging, single-cell RNA sequencing, and human co-culture systems have revealed that epidermal cell transition through distinct functional states and that direct keratinocyte-nerve interactions—including ensheathment and connexin 43-based communication—occur during cutaneous repair (Haensel et al., 2020; Erbacher et al., 2024). Functionally, neural signaling can accelerate re-epithelialization by promoting keratinocyte migration and differentiation, and factors such as GDNF can coordinately regulate Schwann cell activity and keratinocyte behavior, thereby restoring neuro-epithelial coupling (Sudhadevi et al., 2022). Consistent with these observations, animal studies have shown that denervation reduces dermal thickness, disrupts collagen alignment, and delays epidermal reconstruction in wounded skin (Lu et al., 2024; Siu et al., 2025).

**FIGURE 4:**
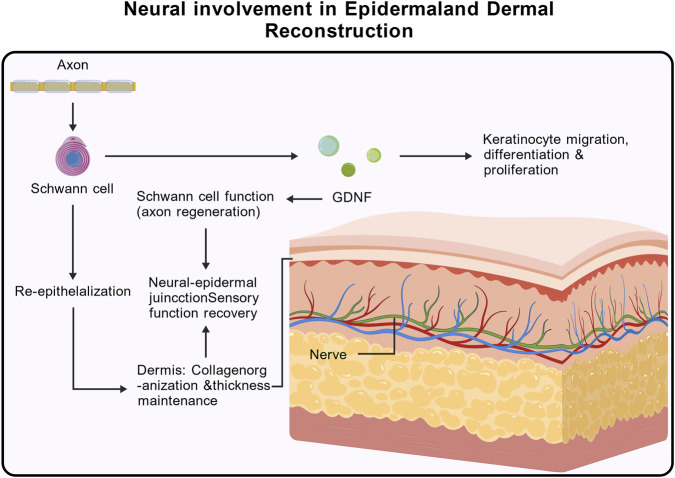
Neural involvement in the reconstruction of epidermal and dermal structures. (Created with BioGDP.com).

Taken together, these observations demonstrate that peripheral sensory nerves regulate wound repair through multiple interconnected mechanisms, including direct factor-mediated signaling, neuro-immune crosstalk, neurovascular coordination, exosome-mediated communication, and the structural reconstruction of the epidermis and dermis. Rather than serving only as information carriers, nerves function as active organizers of regenerative skin repair. This multifaceted regulatory mechanism provides the biological basis for the subsequent design of neurogenic hydrogel systems aimed at restoring both tissue integrity and function (Figure 4).

## 3 Design principles of hydrogel materials and neural functionalization

**FIGURE 5:**
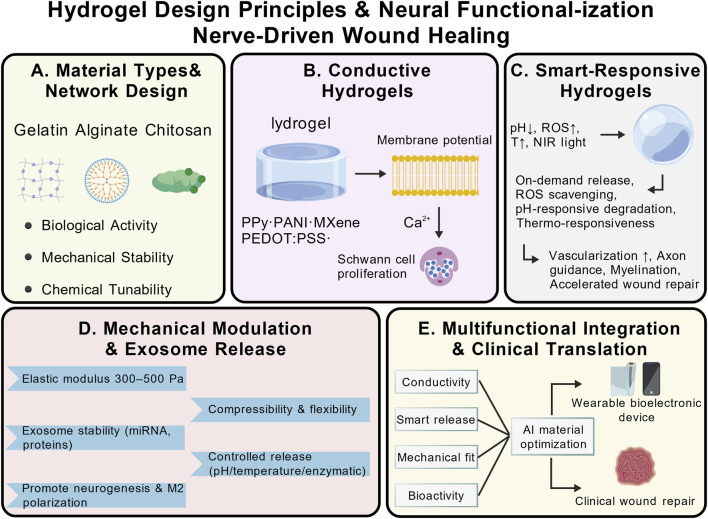
Principles of hydrogel design and neural functionalization, neurogenic wound healing. (Created with BioGDP.com).

Hydrogels are widely used in tissue engineering and regenerative medicine owing to their ability to mimic the ECM alongside their malleability, injectability, and biocompatibility. To meet the specific requirements of neurogenic wound repair, hydrogel design must evolve from a passive scaffold into an active regulatory carrier capable of directing nerve regeneration, modulating the immunological milieu, and promoting angiogenesis, in addition to supplying a wide range of substances. Functionalization strategies typically incorporate exosome delivery, mechanical tuning, stimuli-responsiveness, and electrical conductivity to achieve these outcomes (Figure 5). Based on their composition, hydrogels are categorized into natural, synthetic, and composite types. Natural hydrogels, such as gelatin, HA, alginate, and chitosan, offer inherent biocompatibility and bioactivity, including cell-adhesive motifs that support Schwann cell attachment, axonal extension, and NSC survival. Synthetic hydrogels, such as PEG and PVA, allow for precise control over mechanical properties, crosslinking density, and degradation kinetics, which enables reproducible fabrication and the tunable delivery of NTFs. Composite hydrogels synergistically combine natural and synthetic components to integrate biological recognition with adjustable physical properties, making them particularly advantageous for integrating conductive nanomaterials that mimic the electrophysiological niche of native neural tissue.

### 3.1 The coupled advantages of natural, synthetic, and composite hydrogels

Hydrogels are versatile biomaterials that have advanced significantly through the use of natural, synthetic, and composite fabrication techniques. Each of these approaches offers unique advantages that address specific challenges in tissue engineering and biomedical applications. Natural polymer hydrogels, such as HA, collagen, and gelatin, are prized for their biocompatibility, biodegradability, and ability to mimic the ECM (Xu et al., 2021; Zhao B. et al., 2022). However, the use of these materials in load-bearing applications is frequently restricted by their inherent mechanical weaknesses (Ronca et al., 2018). To overcome these limitations, synthetic polymers such as poly (ethylene glycol) diacrylate (PEGDA) and polyacrylamide are incorporated to enhance mechanical strength and tunability (Thoniyot et al., 2015; Mihajlovic et al., 2022). Composite hydrogels combine the bioactivity of natural polymers with the structural stability of synthetic materials to create hybrid systems with specialized mechanical, chemical, and biological properties (Hao et al., 2017; Rial et al., 2020). Double-network (DN) hydrogels, which consist of two interpenetrating polymer networks, exhibit remarkable toughness and elasticity that make them appropriate for demanding applications such as cartilage repair and 3D bioprinting (Williams et al., 2021; Andrée et al., 2023). Furthermore, the addition of inorganic nanoparticles, such as hydroxyapatite (HAp), to hydrogel matrices improves mechanical characteristics and provides the necessary bioactivity for bone tissue regeneration (Wu et al., 2021; Yadav et al., 2024). These advancements demonstrate the synergistic potential of coupled natural-synthetic-composite hydrogels and enable the construction of multifunctional biomaterials that satisfy the complex requirements of regenerative medicine and beyond. From a clinical translation perspective, the selection of hydrogel components must account for the general pathophysiology of chronic wounds—including impaired angiogenesis, persistent inflammation, and infection susceptibility—as well as the critical role of peripheral nerve dysfunction in delayed tissue repair (Tibatan et al., 2025). In neuro-driven skin regeneration, hydrogels are increasingly designed as active platforms that modulate the neuro-immuno-vascular axis. For instance, composite hydrogels incorporating natural polymers such as gelatin or HA with conductive nanomaterials can mimic the electrophysiological niche of native neural tissue, thereby promoting Schwann cell migration, axonal guidance, and the spatiotemporally controlled delivery of NTFs such as NGF and GDNF. Additionally, the incorportation of reversible dynamic covalent bonds endows the hydrogel network with self-healing properties, which enhances its mechanical resilience under dynamic wound conditions. This is particularly advantageous for dressings applied to mobile anatomical sites where nerve regeneration is actively occurring. These material design strategies align directly with the clinical necessity for long-term, controllable, and patient-friendly treatment modalities that support functional nerve regeneration alongside skin reconstruction.

The methodical combination of materials to achieve specific functional outcomes represents a critical aspect of hydrogel design. Natural polymers, such as HA and gelatin, are often chemically modified with reactive groups, such as methacrylate or catechol, which enable photo-crosslinking or oxidative crosslinking for enhanced mechanical stability (Garreta et al., 2024; Zuo et al., 2024). Such modifications allow for precise control over the microstructure and degradation profile of the hydrogel, in addition to improving its mechanical properties (Giraudo et al., 2020). Conversely, synthetic polymers provide a high degree of tunability regarding swelling behavior, crosslinking density, and stimulus responsiveness (Elkhoury et al., 2021). The integration of these two polymer classes allows the development of hydrogels that combine the mechanical and chemical versatility of synthetic materials with the inherent bioactivity of natural polymers. For example, gelatin methacryloyl (GelMA) hydrogels possess exceptional cell-adhesive and proliferative capabilities while maintaining adjustable mechanical stiffness, and are thus widely used in tissue engineering (Feng et al., 2020; Garcia-Garcia et al., 2024). Additionally, the incorporation of reversible dynamic covalent bonds, such as hydrazone bonds and Diels–Alder adducts, endows hydrogel networks with viscoelastic, injectable, and self-healing properties. These characteristics render such materials ideal for specialized applications in drug delivery and bioprinting (Shu et al., 2024; Shukla et al., 2025).

Hydrogel materials can be categorized into three groups based on their origin: synthetic polymers, such as PEG and PLGA; natural polymers, such as chitosan, gelatin, and alginate; and composite systems consisting of both. Natural materials are rich in bioactive sequences, most notably the arginine-glycine-aspartate (RGD) tripeptide motif. This motif constitutes a key recognition sequence for integrin binding, which supports cell adhesion, migration, and survival. Such properties make these materials ideal for use as nerve guidance channels and for cell encapsulation. In comparison, synthetic polymers, such as PEG and poly (lactic-co-glycolic acid) (PLGA), display precisely controlled cross-linking structures, superior mechanical stability, and reproducibility in processing. Composite strategies, such as systems composed of alginate and PEG, combine natural bioactivity with chemical controllability by varying cross-linking density and functional group types. These hybrid systems can facilitate the chemotaxis of NSCs, support axonal guidance, and enhance overall cellular activity (Cui et al., 2022). Furthermore, the introduction of charge-based or ester bond modifications can enhance the binding affinity and the controlled-release properties of the hydrogels for NTFs.

### 3.2 Conductive hydrogels: constructing biomimetic bioelectric microenvironment

Conductive hydrogels have emerged as a significant class of materials for developing biomimetic electrical signal microenvironments, as they offer a unique combination of electrical conductivity, mechanical flexibility, and biocompatibility comparable to those of biological tissues. These materials have considerable potential for applications in bioelectronics, tissue engineering, and brain interfaces because they can mitigate the mismatch between the soft, hydrated environment of living tissues and the rigid, dry components of conventional electronic devices (Tringides et al., 2023b). By mimicking the ECM while simultaneously enabling electrical signal transduction, conductive hydrogels facilitate cellular growth, differentiation, and communication (Korevaar et al., 2020).

The development of hybrid conductive hydrogels, which integrate conductive polymers, metal nanoparticles, or carbon nanomaterials into hydrophilic polymers, represents a major advancement in this field. Because of its high conductivity and processing versatility, poly (3,4-ethylenedioxythiophene):poly (styrenesulfonate) (PEDOT:PSS) has been utilized extensively as a primary conductive phase within these hybrid matrices (Tringides et al., 2023a; Liu et al., 2024). However, conventional PEDOT:PSS-based hydrogels often face challenges such as mechanical brittleness and phase separation, which can limit their long-term stability and functionality (Asadnia et al., 2016; Xu et al., 2019). To address these limitations, researchers have explored the incorporation of metal nanoparticles, such as silver or tungsten, into the hydrogel matrices. These composites enhance both conductivity and mechanical robustness, allowing for the development of materials that match the viscoelasticity and modulus of biological tissues (Rotenberg et al., 2019; Zhu et al., 2021). For example, hydrogels containing tungsten microparticles have demonstrated the ability to support macrophage growth for up to 5 days, highlighting their potential for use in long-term implantable devices (Zhu et al., 2023).

Another noteworthy development is the creation of supramolecular conductive hydrogels, which achieve self-healing, stress relaxation, and tunable mechanical properties through dynamic covalent bonds and noncovalent interactions. These materials are suitable for use in soft robotics and bioelectronic interfaces given their high conductivity, toughness, and stretchability (Li H. et al., 2025). Research has demonstrated that a polysaccharide-based conductive hydrogel, crosslinked by reversible covalent and noncovalent interactions, can stimulate myoblast myogenic differentiation, indicating that it has significant potential for tissue regeneration (Wang S. et al., 2024). Furthermore, by fine-tuning the network dynamics of these hydrogels, researchers can produce materials with mechanical and electrical properties tailored for specific applications (Ohm et al., 2023).

The application of conductive hydrogels in brain interfaces is particularly significant. Conventional multielectrode arrays composed of rigid metals often fail to accommodate the complex architecture of brain tissues, leading to suboptimal integration and poor signal transduction (Xue et al., 2025). Conversely, conductive hydrogels can be engineered to mimic the electrophysiological and mechanical characteristics of neural tissues, allowing for seamless integration and long-term stability (Chen et al., 2025). For instance, scaffolds composed of porous alginate hydrogels filled with carbon nanomaterials have been developed to support the proliferation and differentiation of neural progenitor cells (NPCs) into astrocytes and oligodendrocytes, enabling the formation of dense, three-dimensional neurite networks (Sun et al., 2024). These scaffolds further enhance cellular differentiation and network formation when exogenous electrical stimulation is applied, offering a physiologically relevant platform for investigating neural development and repair (Ji et al., 2025).

Given the inherent electrophysiological properties of neural tissue, effective regeneration and repair depend on mimicking its endogenous electrical activity. The incorporation of conductive materials such as PEDOT:PSS, MXenes, polypyrrole (PPy), and polyaniline into hydrogels provides the scaffolds with both electrical and ionic conductivity. These functional properties facilitate Schwann cell proliferation, axonal elongation, and nerve impulse transmission via the modulation of cell membrane potential, calcium ion influx, and signaling pathway activation.

Bi et al. developed a conductive hydrogel that combined PEDOT:PSS with an antibacterial and near-infrared-responsive curcumin release mechanism. This multifunctional scaffold significantly improved the local immune microenvironment and promoted nerve regeneration in a diabetic neuropathy model, achieving triple-synergistic control of neural, immune, and conductive aspects (Bi et al., 2025a). Additionally, the incorporation of MXene nanomaterials enhances the overall tensile characteristics and microscopic conductivity of hydrogels, providing a structural foundation for the development of flexible, wearable neural patch materials (Fan et al., 2024b).

The therapeutic potential of integrating materials science with electrophysiology is becoming increasingly evident. Printable conductive hydrogels, when combined with anti-inflammatory drugs, have been shown to simultaneously improve re-epithelialization, collagen remodeling, and the M2/M1 macrophage ratio in diabetic rat wounds, suggesting that a quantifiable synergy exists between conductivity and immunity (Cao et al., 2022). Furthermore, under electrical stimulation, a conductive microtubule hydrogel inspired by the architecture of tubulin “biological wires” can markedly accelerate the closure of diabetic wounds in mice, which occurs within 7 days. This acceleration is accompanied by increased angiogenesis and the upregulation of the expression of nerve growth markers, suggestive of a tri-directional resonance between materials, electrophysiology, and cytokines (Fan et al., 2024c). Stretchable conductive tissue adhesives and nerve-mimetic electroceutical hydrogels demonstrated modulus matching, strong adhesion, and a low immune response within nerve injury and peripheral nerve repair models. These properties support the translatability of compliant electrodes and conformal electrical stimulation in wound treatment (Dhal et al., 2024a). Electrical stimulation can further enhance the regenerative potential of conductive hydrogel platforms. Beyond serving as electroactive scaffolds, these materials can be integrated with bioelectrical stimulation systems to regulate cell behavior and accelerate tissue repair. For example, bioelectric healing-on-a-chip devices enable the simulation of endogenous wound electric fields for the investigation of electrotaxis during wound closure (Zajdel et al., 2021). In these studies, it was shown that electrical stimulation can promote cell migration and improve wound closure under controlled experimental conditions. Importantly, stimulation parameters can be adjusted to optimize therapeutic effects while minimizing excessive electrical stress on cells (Zajdel et al., 2021). These findings support the value of combining conductive hydrogels with tunable electrical stimulation strategies for wound healing and neural repair.

### 3.3 Intelligent responsive hydrogels: adapting to the dynamic changes of the wound microenvironment

Intelligent responsive hydrogels represent a significant advancement in wound care owing to their capacity to adapt to the intricate and shifting wound microenvironment. These systems incorporate specific moieties that detect fluctuations in temperature, redox potential, and pH, as well as enzymatic activity, all of which serve as vital markers for wound healing progression (Liu et al., 2023). Smart hydrogels function as controlled delivery systems that provide flexible drug delivery, cell behavior modification, and tissue regeneration through their ability to mimic natural tissue dynamics (Zhang Y. et al., 2021). For instance, thermoresponsive hydrogels undergo phase transitions to conform to the wound bed and promote drug release, whereas pH-sensitive hydrogels release bioactive molecules in response to the acidic environment of an infected wound (Koetting et al., 2015; Alves et al., 2020). The detection of these environmental changes allows for precise and immediate therapeutic responses, which lead to improved outcomes in wound treatment (Cai et al., 2021). The clinical management of chronic wounds, particularly in diabetic patients, remains complicated by persistent inflammation, dysregulated protease activity, and microbial colonization. However, peripheral nerve dysfunction also critically impairs the neuro-immune-vascular axis essential for coordinated tissue repair. In this context, intelligent responsive hydrogels have emerged as a powerful platform for orchestrating neuro-driven regeneration by reacting to specific pathological cues that directly impact nerve survival and function. These hydrogels incorporate stimuli-responsive components that undergo structural or chemical changes in response to variations in pH, temperature, ROS, or matrix metalloproteinase (MMP) activity—factors intimately linked to both nerve degeneration and regeneration. For instance, ROS-sensitive hydrogels are engineered to release NTFs, such as NGF and GDNF, during peaks of oxidative stress in the wound microenvironment, thereby promoting Schwann cell survival, axonal sprouting, and remyelination while simultaneously dampening neuroinflammation. Similarly, the acidic milieu of infected wounds can be exploited through the use of pH-responsive systems, leading to the release of antimicrobial agents, and, consequently a reduction in the bacterial burden that would otherwise compromise nerve fiber regeneration. Enzyme-responsive hydrogels enable the spatiotemporally controlled delivery of exosomes or immunomodulatory factors in synchrony with the healing cascade, facilitating the transition from a pro-inflammatory to a pro-regenerative microenvironment that supports both axonal extension and vascular ingrowth. These smart features are particularly critical in neuro-driven wound healing, where the precise timing of NTF presentation, immune cell polarization, and vascular maturation is a key determinant of the successful re-establishment of functional nerve networks and the restoration of sensory-motor integration (Tibatan et al., 2025).

The integration of multiple stimuli-responsive functions represents a major advancement in the development of smart hydrogels, addressing the complex physiological changes that occur during wound healing. Combining pH and enzyme-sensitive components allows these hydrogels to undergo targeted degradation when MMPs reach the high levels characteristic of prolonged wound healing processes. These systems incorporate two distinct triggers that initiate the release of anti-inflammatory and antibacterial substances (Hua et al., 2023; Stan et al., 2023). This dual responsiveness not only accelerates the healing process but also protects against complications such as infection and excessive inflammation (Anthis et al., 2024). Furthermore, the incorporation of boronic ester and Schiff base dynamic bonds imparts self-healing properties, ensuring that the materials maintain mechanical integrity and functional longevity in challenging wound environments (Ejeromedoghene et al., 2025; Ho et al., 2025). Smart hydrogels present a significant opportunity for wound care by enabling the design of individualized treatments that adjust their response based on specific wound requirements (Rahmat et al., 2024).

Beyond traditional wound dressings, smart responsive hydrogels serve as platforms for cutting-edge treatment techniques, including gene therapy, growth factor delivery, and stem cell administration. Hydrogels designed to release VEGF in response to hypoxia have been shown to promote angiogenesis and accelerate the healing of chronic wounds (Zhang T. et al., 2024). Tissue regeneration is further improved through the use of temperature-sensitive hydrogels that provide controlled encapsulation and release of MSCs (Rosa et al., 2025). The versatility of smart hydrogels allows for the management of diverse clinical requirements, addressing the specific needs of both acute and chronic wounds. These properties of smart hydrogels ensure that they can be applied across different healing stages, ranging from the immediate treatment of fresh injuries to the management of long-term diabetic ulcer complications (Mohamed et al., 2019).

The wound healing process involves multiple microenvironmental changes, including temperature variations, elevated ROS levels, and pH fluctuations. Intelligent responsive hydrogels facilitate a more precise repair process through controlled biofactor release and gel-state modulation in response to these environmental stimuli. For instance, Dong et al. developed an injectable ROS-responsive hydrogel system based on mPEG-PA-PP, designed to carry H_2_S molecules. In this system, oxidative stress triggers the release of H_2_S, which subsequently supports angiogenesis, immunological polarization, and neuroprotection (Dong et al., 2023). Similarly, thermosensitive hydrogels have shown promise in applications relating to spinal cord injury and peripheral nerve repair. These materials undergo gelation upon reaching human body temperature, a transition that enables the controlled release of NTFs (An et al., 2020).

### 3.4 The coupled strategy of mechanical stiffness regulation and exosome release

The regulation of mechanical stiffness and the controlled release of exosomes are essential for the healing of skin wounds, providing novel therapeutic approaches for improving tissue regeneration. The rigid physical structure of the skin influences cellular behavior through the modulation of exosome release; these nanovesicles function as tissue maintenance agents that enable cell-to-cell communication (Huang et al., 2021; He et al., 2024). Studies have demonstrated that exosomes derived from MSCs enhance wound recovery by modulating angiogenesis, collagen deposition, and inflammatory responses (Gao et al., 2020; Hettich et al., 2020). Notably, the mechanical characteristics of the ECM can influence exosome secretion and bioactivity. Because increased matrix stiffness is associated with myofibroblast activation and the progression of fibrosis, targeting specific mechanosensitive pathways, such as fibroblast activation protein-α, can mitigate these effects (He et al., 2024).

Mechanical stiffness is a primary factor influencing exosome release. Notably, under stretching forces, E-cadherin-mediated cell-cell junctions and FOXO1/KLF4 signaling are disrupted, leading to the loss of tissue regenerative potential (Huang et al., 2021). This mechanotransduction process leads to both enhanced keratinocyte proliferation and the secretion of exosomes containing bioactive molecules, such as microRNAs (miRNAs). Furthermore, E2F1-depleted exosomes derived from adipose-derived stem cells have been shown to enhance wound recovery via miR-130b-5p, which stimulates fibroblast TGF-β signaling, and consequently promotes angiogenesis and collagen production (Yu et al., 2023). Moreover, exosomes containing elevated levels of miR-135a accelerate wound recovery through their ability to block LATS2, thereby influencing cell migration (Gao et al., 2020).

The therapeutic efficacy of exosomes is further enhanced by their ability to influence the physical properties of the surrounding tissue. For instance, exosomes from human bone marrow stromal cells contain bioactive lipids that interact with the CD73 transmembrane enzyme, thereby promoting vascular development and tissue remodeling (Hettich et al., 2020). Because the hydration and mechanical state of the wound bed influence exosome-based treatment effectiveness, optimizing the mechanical microenvironment is crucial for improving therapeutic outcomes (Mijaljica et al., 2023).

The regulation of mechanical stiffness and exosome release can be achieved through two innovative approaches: bioengineered materials and mechanical stimulation. Light-responsive protein fibers provide a novel platform for regulated exosome administration owing to their inherent capacity to undergo reversible changes in toughness and stiffness (Sun et al., 2021). The Hippo signaling molecule TAZ, which controls cell metabolism and function under stress, is activated through mechanosensitive pathways when skin cells experience mechanical stretch (Chakraborty et al., 2021). Integrating exosome therapies with mechanical stiffness control systems represents a promising strategy for enhancing skin wound recovery.

The mechanical characteristics of hydrogels determine how neural cells attach to the material, extend, migrate, and undergo differentiation. NSC development into neuronal cells requires a microenvironment with elastic modulus values ranging from 300 to 500 Pa. This environment provides benefits for controlling miRNA release rates and maintaining exosome structural stability. Fan et al. developed a low-stiffness conductive hydrogel incorporating exosomes derived from bone marrow-derived MSCs. This platform elicited several regenerative effects, including the recruitment of NSCs, enhanced myelination, and macrophage M2 polarization. In a model of spinal cord injury, this approach led to a major improvement in the restoration of neuronal function (Fan et al., 2022). The structural attributes of the hydrogel, namely, its porosity and hydration, determine both exosome loading efficiency and release patterns. Accordingly, the hydrogel structure constitutes a fundamental element that requires integrated design with other system components (Fan M.-H. et al., 2024).

### 3.5 The cutting-edge trend of multifunctional integration

The integration of bioelectronic devices with conductive hydrogels, exosomes, and NTFs into wearable neural modulation systems has yielded promising therapeutic outcomes for diabetic foot ulcers and chronic wounds (Dhal et al., 2024b) (Table 1).

**TABLE 1:** Hydrogel-based materials and their functions in neuro-driven wound healing.

Material/Matrix type	Function	Stimuli-responsive	Release/Delivery mode	Payload	Primary outcomes/Readout indicators	Animal model/Application Scenario	Refs
PQCS/OD/AMNP@Cur (PQCD-A@Cur) Hydrogel	Antioxidant, anti-inflammatory, nerve repair, conductivity	Near-infrared (NIR) light responsiveness	NIR-triggered controlled release of curcumin	Curcumin	Neurogenesis, anti-inflammatory effects, angiogenesis, collagen deposition, wound healing rate	Rat peripheral nerve injury model, infected diabetic wound model	Bi et al. (2025b)
GelMA Hydrogel	Promote diabetic wound healing, antioxidant, antibacterial	—	Sustained release of CGRP	CGRP	Wound healing rate, angiogenesis, collagen deposition, anti-inflammatory effects, cell proliferation	Diabetic mice wound model, potential for clinical translation	Li et al. (2025b)
MT-MAA Hydroge	Conductive dressing for chronic wound healing, angiogenesis, nerve regeneration	ES	Sustained release of growth factors (VEGF, TGF-β, EGF)	MTs, MAA	Wound closure rate, angiogenesis, nerve fiber growth, inflammation control, antibacterial effect, cell proliferation and migration	Full-thickness diabetic wound model in C57BL/6J mice, potential for nerve regeneration and cardiac tissue engineering	Fan et al. (2024c)
Anisotropic nanofiber hydrogel (AFGKLT)	Accelerate diabetic wound healing	Structural anisotropy and VEGF-mimetic peptide	Dual impact of oriented structural and pro-angiogenic signals	VEGF-mimetic peptide	Enhanced multicellular modulation, Improved inter-microenvironmental, Accelerated wound closure and skin tissue regeneration	Diabetic rat model (full-thickness skin wound)	Kim et al. (2025)
QCSMOF-Van (Curcumin-based MOF loaded with Vancomycin and coated with Quaternary Ammonium Salt Chitosan) Hydrogel Matrix	Antimicrobial, Anti-inflammatory, Neuroregenerative, Angiogenic, Tissue Adhesive, Photothermal	Photothermal Responsiveness	Sustained release of Zn^2+^ and Vancomycin Photothermal effect	Vancomycin (Van) Zn^2+^	Neuroregeneration (β3-tubulin expression) Immune Modulation (CD206 and CD86 expression) Angiogenesis (CD31 expression)	Rat Chronic Infected Skin Defect Model/Chronic Wound Treatment	Huang et al. (2022)
GTB hydrogel with NIR irradiation	Enhanced photothermal antibacterial and vascularization	Photothermal effect under NIR irradiation, pH-responsive degradation, glucose-responsive degradation	Release of bioactive ions (Fe3+, Si) and photothermal conversion	TA, Gel-BA, iron-containing bioactive glasses	Significantly enhanced antibacterial activity against *E. coli* and *S. aureus*, Improved angiogenesis and nerve regeneration, Reduced inflammation, Accelerated wound healing with better granulation tissue formation, collagen deposition, and re-epithelialization	Infected full-thickness skin wound model in rats	Hou et al. (2023)
Combination of PFKU/DOXH membrane and GelMA-BioIL hydrogel	Synergistic wound healing through electroactivity and immunomodulation	ROS-responsive degradation and conductivity	Dual impact of DOXH release and electroactivity	DOXH and Bio-IL	Superior wound healing with better epithelialization, angiogenesis, and collagen deposition, Downregulation of ROS and inflammatory factors, Upregulation of M2 macrophage polarization	Diabetic rat model (full-thickness skin wound)	Cao et al. (2022)
Hydrogel incorporated with EGCG@FSG nanoparticles	Multifunctional wound healing platform	pH-responsive degradation and drug release	Release of bioactive compounds (SAB, GOx)	EGCG@FSG nanoparticles	Accelerated DFU healing, Enhanced tissue regeneration, Reduced bacterial load and local inflammation, Promoted angiogenesis and re-epithelialization	DFU model in rats	Gong et al. (2025)
Alginate/Gum Arabic-based Hydrogel with MSNs	Wound healing, tissue regeneration, mechanical reinforcement	—	Sustained release	NGF, Carnosine	Mechanical properties (Young’s modulus), swelling ratio, degradation rate, cytocompatibility, cell proliferation, gene expression (IL-6, TNF-α, NF-κB1, VEGF)	STZ-induced diabetic rats (full-thickness excision wounds)	Keykhaee et al. (2023b)
Silk Fibroin-Based Hydrogel Fibrous Scaffold	Wound healing, bone regeneration, nerve repair	Electrospinning, UV irradiation, metal-ion chelation	Sustained release	Ag+, Mg2+, Zn2+	Cytocompatibility, antibacterial activity, osteogenic and angiogenic potential, nerve regeneration	Rat models for wound healing, bone regeneration, and nerve repair	He et al. (2025)
GelMA hydrogel + VO_2_@ZIF-67 (VZ) + CXCL12 + Lig + Rg1	Synchronous angiogenesis and neuroregeneration; establishes an artificial niche for *in situ* tissue repair	pH-responsive (Co^2+^ release from VZ in acidic wound microenvironment)	Dual release: (1) pH-triggered Co^2+^ release from VZ for angiogenesis; (2) sustained release of CXCL12, Lig, and Rg1 from GelMA hydrogel matrix	Co^2+^, CXCL12, Ligustroflavone, Ginsenoside Rg1	Within 17 days: enhanced CD31^+^ angiogenesis; increased recruitment of CD90^+^ BMSCs; upregulation of nestin and β3-tubulin in regenerated epidermis and dermis; accelerated wound closure; improved collagen organization and skin appendage formation	Full-thickness excisional skin wound model in SD rats	Yuan et al. (2023)
Alginate/gum arabic (AG) hydrogel + mesoporous silica nanoparticles (MSNs) loaded with NGF (SiNGF) + Car	Diabetic wound regeneration; sustained delivery of NGF; anti-inflammatory; pro-angiogenic; promotes re-epithelialization, collagen deposition, and nerve regeneration	Non-responsive (sustained release from MSNs and hydrogel matrix)	Sustained release: NGF loaded into MSN pores and dispersed in AG hydrogel; release profile shows zero-order kinetics with ∼77.9% release over 21 days; carnosine released from hydrogel matrix	NGF (70.55 ng/mL loading), carnosine (5 mg/mL)	Sustained NGF release >21 days; enhanced neurofilament (NF-200) expression in diabetic rat wounds; accelerated wound closure (97% at day 14); increased VEGF and TGF-β expression; improved re-epithelialization, angiogenesis, and collagen deposition; reduced inflammation	STZ-induced diabetic rat model; full-thickness excisional skin wound	Keykhaee et al. (2023c)
CSMA-dAM bilayer hydrogel + SVF-derived exosomes (CSMA-dAM@EXO); CSMA: methacrylated chitosan; dAM: decellularized amniotic membrane; bilayer structure formed via photo-crosslinking	Scar-free healing of diabetic burn wounds; multifunctional repair encompassing anti-inflammation, neurovascular regeneration, collagen remodeling, and anti-fibrosis	Photo-crosslinkable (fabrication process); no exogenous stimuli-responsive release mechanism upon application	Sustained release: SVF-EXO loaded within the CSMA hydrogel layer; rapid release (∼80%) within the first 10 days, followed by gradual release thereafter	Stromal vascular fraction-derived exosomes	*In vitro*: enhanced EPC migration and tube formation; promoted RSC migration; increased PC12 neurite outgrowth; inhibited TGF-β1-induced fibroblast fibrosis (downregulation of α-SMA, SMAD2, COL-1). *In vivo* (diabetic mouse burn wound model): suppressed inflammation during the inflammatory phase; promoted CD31^+^ angiogenesis, β3-tubulin^+^ neurogenesis, and Ki67^+^ re-epithelialization during the proliferative phase; reduced COL-1/COL-3 ratio, decreased α-SMA expression, and improved collagen fiber alignment during the remodeling phase, achieving scar-less healing	STZ-induced diabetic C57BL/6 mice; third-degree burn wound (10 mm diameter, 200 °C for 10 s) on the dorsum, followed by debridement and hydrogel application	Xiao et al. (2025)
Nano-GDY@SH hydrogel (nanosized graphdiyne-loaded sodium hyaluronate hydrogel)	Skin radioprotection via dual-mode physical shielding (low-energy X-ray attenuation) and chemical ROS scavenging	Non-responsive	Topical application; no active release mechanism – the hydrogel acts as a direct protective barrier and radical scavenger	None	Absorbs low-energy X-rays; scavenges broad-spectrum free radicals (OH, O_2_^−^, X-ray-induced ROS); reduces skin edema, ulceration, and inflammation in mice; mitigates pathological lesions; promotes wound recovery and hair regeneration	Mouse low-energy X-ray-induced skin injury model	Xie et al. (2022)
GK@TA gel (ECM-inspired glycopeptide hydrogel), composed of glucomannan grafted with self-assembling KK peptide, TA, and PVA	RSI repair; multifunctional roles including ROS scavenging, M2 macrophage polarization, anti-inflammation, and pro-angiogenesis	Non-responsive	Topical application; the hydrogel acts as an active material directly on the wound site with no exogenous payload release mechanism	None	*In vitro*: scavenging of H_2_O_2_ and DPPH free radicals; reduced intracellular ROS levels in L929 cells after X-ray exposure; decreased γ-H2AX DNA damage foci; enhanced cell viability. *In vivo*: promoted M2 macrophage polarization; reduced pro-inflammatory cytokines; enhanced angiogenesis; reduced epidermal hyperplasia; promoted hair follicle regeneration; improved collagen deposition; accelerated wound healing	Mouse radiation-induced skin injury model	Feng et al. (2023)

QCSMOF-Van,Curcumin-based MOF, loaded with Vancomycin and coated with Quaternary Ammonium Salt Chitosan; PQCS, Polyaniline-grafted quaternized chitosan; OD, oxidized dextran; AMNP, artificial heterogeneous melanin nanoparticles; GelMA, gelatin methacryloyl; CGRP, Calcitonin Gene-Related Peptide; MTs, Microtubules; MAA, methacrylated alginate; ES, electrical stimulation; AFGKLT, anisotropic nanofiber hydrogel; PFKU, polyurethane; DOXH, doxycycline hydrochloride; GTB, Gelatin-based composite hydrogel; TA, tannic acid; Gel-BA, Phenylboronic acid-modified gelatin; EGCG@Fe, Metal-polyphenol nanoparticles; DFU, diabetic foot ulcer; MSNs, Mesoporous Silica Nanoparticles; NGF, nerve growth factor; STZ, Streptozotocin; Lig, Ligustroflavone; Rg1,Ginsenoside Rg1; AG, Alginate/gum arabic (AG); Car, carnosine; RSI, Radiation induced skin injury.

The field is increasingly adopting AI-based methods for material optimization. It has been suggested that a data-driven, function-feedback closed-loop design framework can be established through the use of AI to predict the degree of correspondence between biological effects and material combination parameters (Fan Y. et al., 2024).

The anisotropic structural arrangement of cells enables intercellular coordination mediated through physical cues. Within highly oriented nanofiber hydrogels, the integration of VEGF-mimetic peptides with shape-driven macrophage polarization, Schwann cell maturation, and fibroblast-directed ECM deposition was shown to enhance vascularization. This synergy established a triple-gain effect involving the immunological, vascular, and neural systems in diabetic wounds (Kim et al., 2025). The development of neurogenic hydrogel materials requires a framework that simultaneously regulates structure, bioactivity, and electrochemical properties while delivering biofactors. The evolution of hydrogels into active neuro-wound bridges occurs through three primary advancements: composite strategies involving natural and synthetic polymers, the embedding of conductive components, and the coordination of stimulus-responsive structures with micromechanical and release systems. This emerging pattern accelerates the clinical trajectory for functional skin regeneration.

Multifunctional integration represents the primary trend in skin wound healing, driving fundamental advancements in bioelectronics and regenerative medicine. This transition is being driven by clinical requirements for sophisticated wound management systems that combine diverse functionalities with high performance and personalized care. Additionally, healthcare systems face substantial challenges from recalcitrant wounds, which significantly diminish patient quality of life (Hocking, 2015). The integration of wearable bioelectronics with ECM-based biomaterials shows significant potential by enabling real-time monitoring alongside simultaneous therapeutic delivery. Bioactive tissue sealants, scaffolds, and hydrogels function as ECM-mimetic biologics that replicate the native wound microenvironment, thereby promoting tissue regeneration and enabling the precise delivery of growth factors and pharmaceuticals (Hocking, 2015). Wearable biosensors that monitor microbial, molecular, and physical biomarkers now interface with these materials, establishing closed-loop systems and providing automated wound care through dynamic monitoring (Wang et al., 2022). This comprehensive approach addresses the multifaceted complexities of chronic wound care through streamlined patient management, ultimately resulting in enhanced therapeutic outcomes.

Wearable bioelectronics are becoming essential components of multifunctional wound care, as these systems provide continuous monitoring of critical parameters such as temperature, pH, glucose, and lactate, enabling the detection of infection and the evaluation of healing progress (Shirzaei Sani et al., 2023). Practically, the monitoring interface consists of a wound patch or dressing integrated with biosensors, while microfluidic modules and microneedle arrays facilitate exudate collection and signal acquisition by addressing uneven sample concentrations and weak readouts (Wang et al., 2022).

Machine learning can further enhance the clinical utility of these platforms by analyzing multiparametric sensor data, improving predictive accuracy, and supporting individualized wound assessment and treatment adjustment (Wang et al., 2022). Rather than replacing clinical judgment, machine learning helps clinicians interpret dynamic wound information more efficiently and identify trends that guide therapeutic decisions. In parallel, multiplexed electrochemical biosensor arrays allow for stable and accurate biomarker monitoring, with some systems capable of delivering electrical stimulation or antibacterial and anti-inflammatory interventions. Consequently, these platforms function as integrated monitor-and-treat interfaces for advanced wound management (Shirzaei Sani et al., 2023).

The integration of smart biomaterials with electrical sensors has led to the development of intelligent wound dressings that monitor and adjust wound conditions in real time. These dressings incorporate sensors that track physiological signals through temperature, moisture, and pH measurements. The dressings consist of biocompatible and biodegradable materials, such as chitosan, gelatin, and carboxymethyl chitosan (Yang et al., 2023). By providing immediate data, these dressings assist medical staff in selecting optimal treatment methods while protecting patients from infection and maximizing therapeutic outcomes (Yang et al., 2023). The production of customized wound dressings with specific geometries and functions became possible through the implementation of 3D bioprinting and advanced fabrication methods, including lithography and laser cutting (Wang X. et al., 2023). Furthermore, researchers have developed living Chinese herbal scaffolds that produce photosynthetic oxygen, aiming to enhance chronic wound healing. These bioengineered materials show promise for addressing nutrient and oxygen shortages within the wound bed (Wang X. et al., 2023).

The development of hydrogels and magnetically sensitive fibers provides an advanced system that combines flexible mechanical properties with remote operation and detection capabilities. These materials, which consist of conductive nanoparticles or polymers responsive to external stimuli such as magnetic fields or electrical currents, enable the highly-precise regulation of wound healing processes (Cheng et al., 2025). Studies using photo-inducible hydrogels for the delivery of TGFβ signaling inhibitors have demonstrated effective scarless wound healing in animal models, primarily due to the capacity for temporally controlled substance release (Zhang J. et al., 2021). Furthermore, the integration of carbon nanotubes within hydrogels allows for simultaneous optogenetic stimulation and electrophysiological recording. These systems demonstrate how advanced materials enhance both wound healing outcomes and the performance of neural interface systems (Huang et al., 2024).

## 4 Strategies for the delivery of neurotrophic factors

NTFs regulate various biological processes, including Schwann cell myelination, sensory axonal elongation, the immunological microenvironment, and angiogenesis. NTFs are key modulators of nerve tissue regeneration and overall tissue healing. While basic research has demonstrated the therapeutic value of NTFs, their clinical application remains restricted by unstable molecular structures, short half-lives, inadequate delivery mechanisms, and insufficient local control systems. Consequently, a primary technological challenge at the intersection of materials science and regenerative medicine involves the use of hydrogel substrates to achieve precise positioning, temporal release, and the preservation of NTF biological activity.

### 4.1 Principal neurotrophic factors and their targets

NTFs are essential for neuronal survival, development, and function within the peripheral and central nervous systems. These proteins—including NGF, BDNF, neurotrophin-3 (NT-3), NT-4, and GDNF—operate through specific receptors, driving cellular processes such as axonal development, synaptic adaptation, and neuronal survival (Wang et al., 2015) (Table 2). NTFs function according to specific spatial and temporal requirements, which are necessary for the establishment of neural connections and the maintenance of neuronal activities. For instance, BDNF and GDNF stimulate spinal motor neurons to produce neuregulin-1 (NRG1), enabling the formation of proper axon-target connections in different developmental stages (Wang et al., 2015). These observations indicate that the binding of NTFs to target proteins is essential for the correct neural circuit formation and function.

**TABLE 2:** Neurotrophic factors and their roles in wound healing and Nerve regeneration.

Key factor	Receptor	Main target cells	Stage of action	Main effect	Representative model/Result	Delivery/Intervention platform	Refs
CGRP	RAMP1-CALCRL	Neutrophils, Macrophages	Proliferative stage	Promotes efferocytosis and M2-like polarization via TSP-1	eCGRP delivery reduces inflammatory markers and increases anti-inflammatory markers in diabetic wounds	Local delivery of eCGRP in diabetic mice	Lu et al. (2024)
CGRP	CGRP receptor (RAMP1)	Macrophages, Endothelial Cells	Inflammatory and Proliferative stages	Promotes M2 macrophage polarization, Enhances endothelial cell proliferation and migration, Reduces inflammation, Promotes angiogenesis	CGRP-KO mice: Delayed wound healing, Reduced M2 macrophages, Decreased angiogenesis. GelMA-CGRP hydrogel: Accelerated wound healing in diabetic mice, Increased neoepithelium growth, Enhanced collagen deposition	GelMA-CGRP hydrogel: Sustained release of CGRP, Good biocompatibility, Antioxidant and antibacterial properties	Li et al. (2025b)
CSF1, BMP2	CSF1R, BMPR	Macrophages, Neurons	Inflammatory stage, proliferative stage	Promotes macrophage recruitment and polarization to M2 phenotype, Enhances nerve regeneration and wound healing	Macrophage depletion wound model: Delayed wound healing, reduced nerve regeneration. CSF1 treatment accelerates wound healing by promoting M2 macrophage polarization. BMP2 treatment accelerates wound healing by promoting nerve regeneration	Local delivery of CSF1 via subcutaneous injection in denervated wound model. Local delivery of BMP2 via subcutaneous injection in macrophage depletion wound model	Wang et al. (2024a)
NGF	TrkA, p75NTR	Keratinocytes, Fibroblasts, Inflammatory Cells	Proliferative and Remodeling Phases	Promotes skin regeneration, angiogenesis, collagen synthesis, and nerve innervation	*In vitro*: Increased VEGF expression, improved cell migration and proliferation. *In vivo*: Enhanced wound healing in diabetic rats, increased nerve fiber regeneration	Immobilized in mesoporous silica nanoparticles (MSNs) and incorporated into alginate/gum arabic hydrogel	Keykhaee et al. (2023a)
NT-3	TrkC	Sensory neurons, endothelial cells, fibroblasts, keratinocytes	Wound healing and re-vascularization	Promotes wound healing, angiogenesis, fibroblast function, keratinocyte proliferation	Rat model of diabetic foot injury: SCS therapy increased NT-3 levels, improved wound healing, and increased vascular density	SCS therapy and recombinant NT-3 administration	Liu et al. (2025)
BDNF	TrkB	Neurons, endothelial cells	Wound healing	Modulates endothelial cell function, promotes nerve regeneration	Rat model of diabetic foot injury: BDNF levels remained unchanged after SCS therapy	SCS therapy	Liu et al. (2025)
Semaphorin 3A	Plexin A1	Macrophages	Early Inflammatory Phase	Promotes M2 polarization of macrophages, reduces M1/M2 ratio	BMDMs cultured in sensory neuron-derived conditioned medium show increased M2 markers	Sensory nerve-mediated immunoregulation	Xu et al. (2025)
CGRP	CGRP Receptors	Sensory Neurons, Macrophages, Adipocytes	Early Stage of Tissue Repair	Induce regeneration, counteract scar healing effect of opioids	scAT resection model; CGRP administration induces regeneration, morphine + CGRP allows regeneration with analgesia	Subcutaneous injection of CGRP	Rabiller et al. (2021)
CGRP	RAMP1	Neutrophils, Monocytes, Macrophages	Early and Late Stages of Wound Healing	Promotes M2 polarization of macrophages, accelerates wound healing	*In vitro* assays show CGRP promotes M2 polarization and reduces pro-inflammatory cytokines (TNF-α, IL-1, IL-6) while increasing anti-inflammatory IL-10	PEG/PLMA/CGRP hydrogel	Pi et al. (2025)
Curcuin	​	Macrophages, Schwann Cells, Endothelial Cells	Throughout Wound Healing	Antioxidant, Anti-inflammatory, Promotes Schwann Cell Proliferation and Myelination, Enhances Angiogenesis	*In vitro* and *in vivo* assays show reduced oxidative stress, increased Schwann cell proliferation, enhanced macrophage polarization towards M2 phenotype, improved angiogenesis	PQCD-A@Cur hydrogel with Near-Infrared irradiation	Bi et al. (2025b)

TrkA, Tropomyosin-related kinase A; p75NTR, p75 Neurotrophin Receptor; SCS, spinal cord stimulation; NT-3, Neurotrophin-3; BDNF, Brain-Derived Neurotrophic Factor; BMDMs, Bone marrow-derived macrophages; CGRP, Calcitonin Gene-Related Peptide; scAT, subcutaneous adipose tissue; RAMP1, Receptor Activity Modifying Protein 1.

NTFs exert their complex signaling functions via the activation of multiple intracellular pathways, including the MAPK, PI3K-Akt, and Jak-STAT cascades. For example, the activation of TrkB-MAPK and TrkB-PI3K signaling by BDNF results in increased NRG1 mRNA production, a process associated with the formation of proper axon-target connections. GDNF stimulation similarly activates these pathways across various physiological contexts, illustrating that NTF signaling consists of both specific and redundant mechanisms (Wang et al., 2015). The BDNF-induced expression of NRG1 exhibits distinct temporal patterns, reaching peak levels at 4 h of induction before rapidly declining. This indicates that the successful completion of NTF-mediated events depends on precise timing. Such temporal regulation prevents the formation of aberrant neural connections by limiting NTF signaling to axons that establish stable links with their targets. Furthermore, NTF proteins are subject to additional regulatory layers through post-translational modifications, such as the protein kinase -mediated release of NRG1 from axons (Wang et al., 2015). These findings demonstrate that the accurate regulation of neuronal development and function relies on multiple levels of NTF signaling regulation.

NTFs represent significant therapeutic targets for neurodegenerative diseases and nerve injuries owing to their essential roles in neuroprotection and regeneration. Research has shown that both BDNF and GDNF can restore NRG1 expression following limb bud removal, reflecting their potential for preserving neuronal viability and function after tissue injury (Wang et al., 2015). NTF efficacy appears most pronounced at the peak of tissue damage, provided surrounding cells remain viable. In murine models of Parkinson’s disease, GDNF exhibits more consistent neuroprotective properties than either CDNF or MANF; however, these NTF family members offer distinct advantages depending on the specific treatment site and the degree of tissue damage (Cordero-Llana et al., 2015). The delivery method and the use of combination strategies are critical for maximizing therapeutic benefits. Specifically, studies using Parkinson’s disease models have demonstrated that CDNF and MANF combination therapy provides superior neuroprotection and functional recovery compared to the administration of either factor alone (Cordero-Llana et al., 2015). These findings indicate that a comprehensive understanding of both the precise mechanisms and the environmental conditions governing NTFs is necessary for the development of effective clinical treatments.

NTFs are central to synaptic plasticity and the development of neural connections, rendering them the focus of significant scientific investigation. For example, NRG1 reduces hippocampal long-term potentiation, whereas BDNF promotes this process. This relationship demonstrates that NTFs can mediate opposing outcomes during synaptic activity (Wang et al., 2015). Furthermore, the roles of NTFs in synaptic plasticity are dependent on neuronal activity levels (Wang et al., 2015). The production of BDNF and NRG1 molecules depends on activity patterns within neuromuscular junctions and the central nervous system, and the regulatory mechanisms governing the operation of diverse neural circuits in both systems are identical (Wang et al., 2015). Consequently, the activity-dependent regulation of NTFs allows for the development of synaptic plasticity and neuronal connectivity, both of which are essential for learning and memory.

Beyond their roles in development and plasticity, NTFs are involved in the pathophysiology of various neurological and psychiatric conditions. It has been shown that schizophrenia, depression, and chronic pain are associated with altered levels of BDNF, NGF, and GDNF, making these factors potential biomarkers and therapeutic targets in these conditions (Malfait et al., 2020). Notably, models of chronic pain have identified NGF and BDNF as key factors in the development of the condition (Malfait et al., 2020). Additionally, the dysregulation of glutamatergic and dopaminergic signaling pathways, central to the pathophysiology of schizophrenia, is correlated with changes in NRG1 and BDNF levels (Ricci et al., 2024). These findings underscore the importance of studying NTFs to understand disease progression and develop targeted therapies.

The therapeutic potential of NTFs is attributable to their neuroprotective and regenerative effects, as demonstrated primarily in models of neurodegenerative disease and nerve damage. For instance, BDNF and NT-3 stimulate axonal growth in peripheral nerve injury models, while GDNF provides more consistent protection in models of Parkinson’s disease (Cordero-Llana et al., 2015). Despite this potential, the clinical translation of NTF-based therapies remains restricted by stability and targeted delivery issues. The therapeutic utility of factors such as GDNF, CDNF, and MANF is often constrained by inadequate tissue penetration and variable efficacy based on the delivery site and lesion severity (Cordero-Llana et al., 2015). Overcoming these obstacles necessitates the development of innovative delivery platforms, such as vectors for gene therapy and nanotechnology-based carriers.

NTFs have several functions, which serve to preserve neuronal integrity, support synaptic plasticity, and mitigate disease progression. These molecules act as essential regulators of neuronal survival, development, and physiological function. The maintenance of neural circuit function requires NTF signaling through a multi-level control system that governs both the formation and the operational state of neuronal connections. Because NTFs are fundamental to neuronal protection and regeneration, they remain versatile therapeutic targets for addressing the pathophysiology of neurodegenerative diseases, nerve injuries, and psychiatric disorders.

### 4.2 Multi-factor hydrogel platforms for NTF delivery

While Section 3.3 discussed the design of smart responsive hydrogels, this section focuses on the integration of responsiveness with additional regulatory components, such as exosomes, to construct multi-factor platforms for NTF delivery. Responsive hydrogel platforms have emerged as important carriers because they can adapt to the changing wound microenvironment and support controlled release. For instance, ROS-responsive systems can regulate local oxidative stress while improving the regenerative microenvironment. The mPEG-PMet-based H_2_S-releasing system reduces ROS levels and promotes neurovascular tissue development in injured areas (Dong et al., 2023). Thermosensitive systems also contribute to controlled delivery; PNIPAM hydrogel undergoes sol-gel transition at 37 °C, enabling *in vivo* gelation and the sustained release of encapsulated NTFs (An et al., 2020). Hydrogel-assisted exosome delivery provides an additional strategy for multi-factor regulation. Combining NTFs with exosome-containing hydrogels can improve platform stability and regulatory capacity.

When incorporated into decellularized matrix hydrogels, neurogenic exosomes loaded with miR-124 support spinal cord injury repair (Wang K. et al., 2024). Because of their biocompatibility and structural flexibility, hydrogel-exosome composite systems have become useful platforms for the sustained release of neurofunctional elements. Multi-factor hydrogel platforms integrate controlled release, microenvironmental responsiveness, and exosome-assisted regulation to support neuroregeneration. The role of conductivity and electrical stimulation in neuroregenerative hydrogels is discussed in detail in Section 3.2.

The clinical applicability of these multi-factor delivery platforms is supported by recent advances in the standardization and scalability of hydrogel-based systems. For instance, alginate- and gelatin-based hydrogels have been successfully employed as carriers for NTFs, neurogenic exosomes derived from Schwann cells or NSCs, and immunomodulatory agents. Preclinical models of diabetic wounds—where peripheral neuropathy is a critical pathological feature—have demonstrated that these systems improve axonal regeneration, Schwann cell migration, and neurovascular coupling (Tibatan et al., 2025). A key trend observed in the literature is the increasing complexity of hydrogel formulations, which are evolving from simple passive dressings toward multifunctional systems. These systems integrate neuro-specific cues, such as the controlled release of NTFs, conductive components for electrophysiological mimicry, and immune-modulating exosomes, alongside supportive functionalities such as antibacterial agents, oxygen-generating components, and self-adapting structural features. This evolution directly addresses the clinical need for integrated therapies capable of simultaneously restoring nerve function, modulating the immune microenvironment, and promoting vascularization in chronic wounds. Importantly, the successful translation of such neuro-driven hydrogel platforms requires sophisticated material design as well as robust manufacturing processes, reproducible quality control, and clear regulatory pathways tailored to combination products. These challenges are being progressively addressed through interdisciplinary collaboration between material scientists, clinicians, and regulatory experts.

### 4.3 Research directions

Bionic design provides an innovative approach to the complex challenges of tissue regeneration and functional restoration, particularly for neural-driven skin wound healing applications. This field focuses on advanced solutions that accelerate wound recovery and restore missing sensory and motor functions through the integration of biological knowledge, materials science, and brain engineering principles. The convergence of these disciplines has enabled the development of three technologies with significant clinical potential: wearable skin structures, brain interfaces, and biohybrid devices.

The most significant breakthroughs involve wearable skin constructions that replicate human skin structure and operational behavior. These designed structures demonstrate enhanced mechanical properties, targeted cell arrangement, and increased ECM production (Pappalardo et al., 2023). The vascular network present in these constructs supports tissue integration, which simplifies surgical procedures and improves aesthetic outcomes (Pappalardo et al., 2023). Furthermore, the prevascularization of these constructs before transplantation addresses a primary barrier to graft survival in wounds involving exposed bone or tendons (Pappalardo et al., 2023). This approach provides customized solutions for difficult-to-treat areas, such as the hands of patients with epidermolysis bullosa, and supports future applications in composite tissue transplantation and facial reconstruction (Pappalardo et al., 2023).

Neural-driven wound healing technology has enabled the development of new treatment methods for patients with severe skin injuries who have lost sensory and motor functions. By combining neural interfaces with bioengineered skin substitutes, these systems detect external signals and transmit sensory information to neural pathways. For example, the development of fully implantable wireless tactile sensory systems allows patients with permanent mechanoreceptor damage to perceive tactile sensations (Kang et al., 2024). These systems employ CFAS artificial skin, which incorporates collagen and fibrin to support wound healing, while hydrogel coatings on the neural interface electrodes minimize foreign body reactions (Kang et al., 2024). Such technology transforms touch into electrical signals that activate peripheral nerves, offering a pathway for patients to regain environmental awareness and movement (Kang et al., 2024).

The biohybrid neural interface represents a significant innovation, combining flexible electrode arrays with myocytes derived from induced pluripotent stem cells to enhance neural recording accuracy and functionality (Rochford et al., 2023). This technique establishes a regulated connection between implanted cells and existing neural networks, addressing common neurotechnological challenges such as tissue scarring and signal degradation (Rochford et al., 2023). These biohybrid devices maintain functionality for several months when integrated with animal tissues, demonstrating potential for treating peripheral nerve injuries (Rochford et al., 2023). Furthermore, the development of composite regenerative peripheral nerve interfaces provides patients with sensory feedback and enhanced motor signals, enabling high-accuracy prosthetic control (Svientek et al., 2020).

The role of conductivity and electrical stimulation in neuro-driven hydrogel design is systematically addressed in Section 3.2. Future research should prioritize the integration of these electroactive platforms with emerging technologies—including AI-driven material optimization, closed-loop bioelectronic control, and multimodal therapeutic delivery—to achieve precise, adaptive, and personalized tissue repair.

A primary objective in neuro-epidermal junction reconstruction involves addressing peripheral nerve and epidermal damage through the development of structures that provide differential release rates and responsive microenvironments to guide the simultaneous migration of nerve terminals and keratinocytes (Sudhadevi et al., 2022). AI-assisted design of customized factor release profiles, using machine learning algorithms to forecast the relationship between biological responses and material parameters, allows for the identification of optimal combinations of NTFs and hydrogel characteristics (Fan Y. et al., 2024).

**FIGURE 6:**
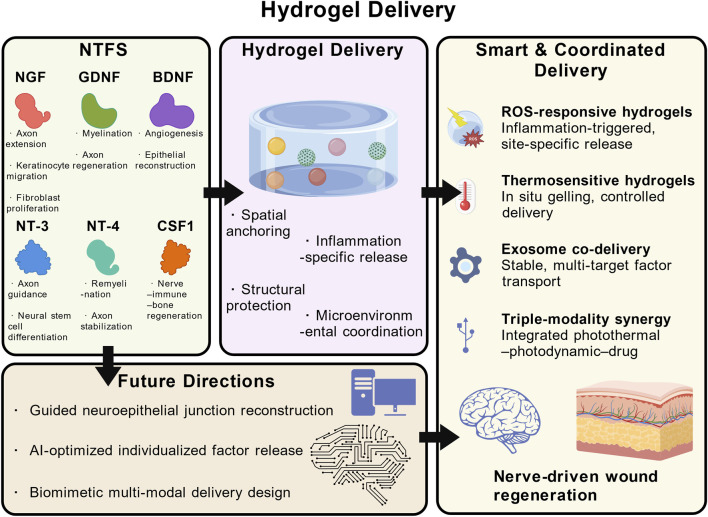
Hydrogel Delivery (Created with BioGDP.com).

Effective neurogenic wound repair requires the precise distribution of neurotrophic agents. Current hydrogel research enhances NTF therapeutic efficiency through the development of advanced drug release systems, protection of biological structures, and diverse delivery methods. The field of neurogenic intelligent materials is approaching clinical readiness as researchers combine exosome systems with electrical conductivity and responsive architectural structures. The development of bionic designs for neural-driven skin wound healing will likely proceed through systems that unite complex cellular therapies with sensory restoration and active wound treatment. Achieving complete skin regeneration necessitates the development of wearable skin structures that include skin appendages, pigmentation, and functional immune systems (Pappalardo et al., 2023). Furthermore, combining machine learning algorithms with wearable bioelectronic systems could establish real-time monitoring, enabling personalized treatments and enhanced system operation (Wang et al., 2022). The scalability of these technologies for treating extensive wounds and complex injuries resulting from burns or trauma demonstrates their significant potential for the future of tissue engineering (Pappalardo et al., 2023) (Figure 6).

## 5 Constructing a validation system for neuro-modulated wound repair

Establishing animal models with high reproducibility, resolution, and analyzable mechanisms is crucial for advancing the understanding of the material-nerve-tissue ternary interaction. These models are essential for mechanism validation and clinical translation, particularly as research on the mechanisms of neuro-modulated wound repair becomes more comprehensive. Numerous animal models have confirmed the inhibitory effect of denervation on wound healing, demonstrating how sensory nerves directly coordinate the recovery process. Consequently, these models serve as vital tools for investigating the *in vivo* performance of neurofunctional hydrogel systems within living biological systems. Although this section focuses on animal models, these validation systems are essential for assessing the *in vivo* performance of neurogenic hydrogels and advancing them toward clinical translation. These additions serve to explicitly link Sections 2, 5 to the overarching theme of hydrogel-based neural engineering without requiring major structural changes to the manuscript. We sincerely hope that these modifications will address your concern. Thank you again for your valuable suggestion.

### 5.1 Model types and classification

The denervated mouse model serves as a critical tool for neurological and musculoskeletal research, enabling the study of mechanisms associated with nerve damage, muscle atrophy, and regenerative growth. These research models are categorized according to the type of nerve injury, the anatomical location of denervation, and the duration of the denervation process, providing specific benefits for investigating different biological systems and disease states.

Acute models allow for the investigation of immediate nerve injury responses through rapid transection or ligation procedures. For example, the sciatic nerve ligation model in C57BL/6J mice facilitates the study of acute-phase responses, including hypoxia, angiogenesis, and macrophage activation. This model is characterized by rapid muscle deterioration and genetic changes that become apparent within the first few days following injury (Sato-Yamada et al., 2025). In contrast, chronic denervation models involve sciatic nerve resection over extended periods. These models allow researchers to examine the long-term effects of denervation, such as the development of muscle fibrosis, Schwann cell senescence, and the inhibition of axonal growth (Fuentes-Flores et al., 2023). By utilizing these models, scientists can evaluate therapeutic methods aimed at minimizing permanent tissue harm and gain insight into the physiological barriers that prevent patients from regaining motor function after chronic nerve damage.

Partial denervation models, such as the spared nerve injury model, require the ligation of specific nerve branches while leaving others intact. This method enables the study of neuropathic pain by creating conditions that match the nerve damage occurring in diabetic neuropathy and other clinical cases (Segelcke et al., 2023). In contrast, the tibial nerve transection model represents a complete denervation model, establishing an extreme injury condition for the study of muscle atrophy, failed reinnervation, and neuromuscular junction degeneration (Ehmsen et al., 2019). These models function as fundamental scientific instruments for investigating nerve regeneration treatments, muscle healing processes, and the natural progression of tissue deterioration.

Denervation models can also be categorized according to the anatomical location of the injury. The spinal cord injury model exhibits muscle denervation because the muscles located below a contusion or transection site lose their neural connections. Researchers use this model to study spinal cord repair mechanisms, including axon regrowth, motor function recovery, and Schwann cell-mediated healing (Borges et al., 2018; Kakuta et al., 2019). Additionally, the pelvic floor denervation model resulting from pudendal nerve damage serves as a tool for investigating pelvic floor dysfunction and the therapeutic potential of MSCs for nerve tissue regeneration (Zhang et al., 2023). Such site-specific models allow for the examination of denervation effects that vary across different anatomical regions.

Research using conditional knockout mice and other genetic models helps scientists understand the molecular mechanisms that occur when nerves disconnect from muscles. The Tie2Cre; R26RTd-tomato mouse model enabled the study of angiogenesis in denervated muscle, revealing that macrophages play essential roles in vascular remodeling processes (Sato-Yamada et al., 2025). The TRPC3 knockout mouse model serves as a research tool for investigating how TRPC3 channels affect the development of spreading depression, a phenomenon that occurs after mechanical injuries and leads to nerve damage and neuroinflammatory responses (Zheng, 2023). These genetic models allow scientists to precisely control particular molecular pathways, providing a clearer view of how specific cells and molecules react to denervation.

### 5.2 Directions for model optimization

The development of denervated mouse models that accurately simulate human conditions and improve treatment outcomes requires focusing on three primary areas: precision, reproducibility, and translational utility. Standardizing surgical techniques and injury protocols is vital for reducing variations in denervation severity and healing patterns. For instance, research using neonatal mice with clip-generated compression injuries has indicated that the force, duration, and speed of compression must be strictly controlled to ensure consistent tissue damage (Züchner et al., 2016). Furthermore, reinnervation can now be monitored in real time through minimally invasive methods that combine implantable electrodes with ultrasound imaging, thus reducing the reliance on end-point assessments (Lowe et al., 2024).

Investigating the molecular and functional changes in denervated muscle necessitates the use of modern technologies, including optogenetics and genomic analysis. Optogenetic stimulation provides a novel approach to studying muscle atrophy and resilience, as this method allows for the control of muscle contraction independently of neural signals (Vajtay et al., 2019). Additionally, transcriptomic analysis of denervated muscles in mouse whisker pad studies has identified specific genes that enhance muscle performance, offering potential targets for therapeutic applications (Vajtay et al., 2019). Integrating these methods with high-throughput phenotyping and genomic selection techniques will likely accelerate the discovery of genetic elements that govern muscle recovery and the rate of reinnervation (Da Silva et al., 2025).

Findings from murine models often fail to translate directly into human clinical applications, necessitating the use of larger animal subjects such as rats. Rats offer distinct advantages for investigating complex neural abnormalities and evaluating therapeutic interventions because of their superior nerve dimensions and regenerative capacities (Hakkı Altuntaş et al., 2024). The implementation of standardized behavioral and electrophysiological protocols, such as gait analysis and retrograde neuronal labeling, would enhance the precision of functional outcome measures in denervated models (Hakkı Altuntaş et al., 2024). In addition to technical refinements in model development, future research should prioritize using these validated systems to test novel therapies that integrate neurogenic hydrogels with stem cell or NTF delivery. Section 6 provides comprehensive analyses of these clinically oriented strategies, specifically regarding the incorporation of hydrogel platforms to support medical translation.

## 6 Clinical application and translation of neurogenic hydrogels in tissue repair

Research into neuro-driven hydrogels for tissue repair has gained prominence because these materials demonstrate potential for establishing environments that resemble native neural tissue for successful tissue regeneration. Although most relevant reviews to date have focused on hydrogels for cardiac, osteochondral, and skin tissues (Hasan et al., 2015; Singh et al., 2018), current evidence suggests that successful neural regeneration necessitates the integration of neuro-specific properties to achieve optimal neural tissue recovery.

Supramolecular adhesive hydrogels for tissue engineering applications have been extensively reviewed regarding their utility in neural repair, specifically for their capacity to establish biomimetic networks that adapt to physiological conditions (Zhao Y. et al., 2022). These materials leverage non-covalent interactions to achieve self-healing capabilities, a feature essential for neural regeneration as it allows the matrix to respond to both mechanical and biochemical signals (Shahriari et al., 2023).

Self-healing hydrogels demonstrate a capacity to repair defects in neural and other tissue types by restoring structural integrity after an injury. These materials support novel strategies for neural tissue regeneration, given that they fulfill the necessary conditions for neural cell growth (Shahriari et al., 2023). Furthermore, the mechanical characteristics of these hydrogels require optimization for neural applications. Specifically, matching the compliant nature of neural tissue is necessary to support cell survival and differentiation processes (Wang et al., 2023b).

The neuro-regenerative potential of hydrogels can be increased through the integration of biological cues, such as growth factors and bioactive molecules. Hydrogels containing growth factors have been used to heal cutaneous wounds, and this approach now informs the development of neural tissue engineering methods that support axonal development and neural network formation (Sapru et al., 2021). Biopolymeric hydrogels that respond to neuro-specific signals enable tissue regeneration by supporting neural cell growth and maturation (Sontyana et al., 2018).

Furthermore, the capacity of HA-based hydrogels to function as dynamic and flexible materials makes them suitable for neural repair, as they create an environment that resembles the native neural ECM. These hydrogels demonstrate an ability to detect biological signals, which assists in tissue connectivity and the restoration of functional capabilities (Zhang M. et al., 2024). The development of hydrogels for neural repair depends on two essential design principles: supramolecular strategies and the incorporation of bioactive cues (Zhao Y. et al., 2022; Politrón-Zepeda et al., 2024).

### 6.1 The prospect of AI-material collaborative design

The integration of AI with neuro-driven hydrogels establishes an innovative tissue repair system that addresses complex challenges in neural tissue restoration and soft tissue molding. AI can process large data sets to identify optimal hydrogel formulations, which enables the development of biomaterials that reproduce the biological signals present in native tissues (Cai et al., 2021). Neuro-driven hydrogels that respond to neural signals and environmental changes demonstrate an outstanding capacity to direct cell migration and angiogenesis, which supports the development of functional tissues (Wertheim et al., 2022). Furthermore, the combination of VEGF-mimetic KLT peptides and hydrogels results in enhanced pro-angiogenic effects that aid in the treatment of brain injuries and the establishment of neurovascular regeneration sites (Rosaming et al., 2022). Notably, NSCs differentiate into neuroglial cells when cultured on electrically charged hydrogels containing cationic and anionic monomers, thus contributing to brain parenchyma reconstruction in traumatic injury models (Lu et al., 2019). In practical terms, the integration of machine learning with hydrogel design functions through a closed-loop system that combines experimental data, predictive modeling, and clinical feedback. Experimental data, including hydrogel composition, mechanical properties, degradation kinetics, and biological outcomes, are used to train predictive models using algorithms such as random forests or neural networks. These models establish quantitative structure-activity relationships and enable the generation of optimized hydrogel formulations through iterative feedback loops. Functioning as a clinical decision-support tool, the system allows clinicians to input patient-specific wound parameters. The model then recommends tailored hydrogel compositions, including optimal NTF combinations, exosome loading, and conductive components, which reduces trial-and-error in treatment selection. For monitoring and treatment, a proposed interface combines wearable biosensors with a cloud-based AI platform. Wearable sensors continuously monitor wound biomarkers, and the AI model analyzes these data in real time, adjusting therapeutic delivery via smart hydrogels or wearable bioelectronic devices. The clinician receives visualized reports and treatment recommendations through a secure digital dashboard, enabling remote patient management and individualized therapy.

Researchers employ AI technology to develop hydrogel materials with enhanced properties while predicting the behavior of these materials in biological environments. AI algorithms generate personalized tissue repair solutions through the analysis of hydrogel degradation patterns, mechanical properties, and biochemical indicators (Hasan et al., 2015). AI-driven models determine the optimal growth factor and peptide concentrations for hydrogels to ensure controlled drug release at specific injury sites (Tanikawa et al., 2023). In the context of spinal cord injury repair, injectable hydrogels are employed to establish interfacial bonds to support tissue structure; these materials promote the polarization of macrophages into pro-regenerative phenotypes, alongside angiogenesis and neurite extension (Hsu et al., 2019). The development of adaptive microporous hydrogels (AMHs) was achieved through AI technology, which enabled the creation of scaffolds that distribute growth factors for cell migration, resulting in substantial repair of peripheral nerve defects through axonal growth (Xiouras et al., 2022).

AI technology has enabled the development of hydrogels that respond to the natural biological sequence of inflammation, repair, and reconstruction during soft tissue remodeling. These hydrogels incorporate MMP-sensitive and chemokine-like peptides, which allow the scaffolds to detect environmental changes and recruit cells for colonization and functional development (Sounderajah et al., 2025). Furthermore, thiol-ene click reaction-based hydrogels function as inflammation-sensitive materials; their programmed degradation leads to the release of chemokines that direct cell migration and create surfaces mimicking natural tissue for wound recovery (Obermeyer and Topol, 2021). In one study, a staged approach was employed to achieve improved collagen formation, angiogenesis, and tissue perfusion (Howell et al., 2024).

AI technology was reported to facilitate the delivery of therapeutic agents and stem cells to infarcted cardiac tissues. The predictive capabilities of AI models are leveraged to determine the hydrogel mechanical characteristics and degradation behaviors necessary to satisfy the requirements of the cardiac microenvironment (Bi et al., 2019). Additionally, the injection of hydrogels containing stem cells and cytokines, such as SCF and SDF, improves cell survival rates, tissue integration, and differentiation, leading to enhanced treatment outcomes for myocardial infarction (Eaneff et al., 2020).

While AI-driven hydrogel technologies are increasingly available as commercial solutions, healthcare personnel encounter significant challenges when integrating these systems into medical facilities. The development of safe and effective biomaterials necessitates addressing three primary obstacles: unbalanced datasets, algorithmic bias, and the requirement for testing within authentic clinical environments (Rozera et al., 2025). Furthermore, monitoring hydrogel degradation and tissue ingrowth requires the synchronization of AI with multimodal imaging systems to allow for real-time observation (Zeltzer et al., 2025). Dual-channel fluorescence imaging techniques enable the simultaneous monitoring of scaffold breakdown and tissue healing in brain injury studies, which assists in evaluating the overall effectiveness of hydrogel treatments (Varghese et al., 2024).

### 6.2 The application of exosome-incorporated neurogenic hydrogels in tissue repair

The integration of exosome-based therapies with neuro-driven hydrogels establishes an innovative approach to tissue repair, offering potential for treating complex injuries affecting both the nervous and musculoskeletal systems. Exosomes function as natural nanovesicles that facilitate intercellular communication through the delivery of bioactive molecules, including proteins, lipids, and nucleic acids, to target cells (Cheng and Hill, 2022; Eisenstein, 2022). This process alters cellular behavior and promotes tissue regeneration. The efficacy of exosome delivery increases when combined with hydrogel scaffolds, as these materials allow for the controlled and sustained release of the vesicles within living tissues (Li et al., 2021). These systems achieve optimal results in neural repair because the hydrogels form a structure mimicking the ECM, which supports nerve cell growth and maintains tissue defense functions (Bajic et al., 2024). Specifically, HA-based hydrogels outperform collagen-based alternatives in supporting neural growth and protection by more accurately replicating the native neural ECM (Bajic et al., 2024). Additionally, the mechanical and electrical characteristics of these hydrogels, such as stiffness and conductivity, can be precisely adjusted to direct NSC differentiation and macrophage polarization, processes that are essential for tissue healing (Guan et al., 2024).

The development of electrically conductive hydrogels represents a breakthrough in the field, as these materials preserve tissue architecture while establishing electrical pathways that duplicate the properties of neural tissue (Guan et al., 2024). These hydrogel matrices demonstrate excellent potential for the treatment of spinal cord injury through their capacity to incorporate M2 macrophage-derived exosomes (M2-Exos), which enhance nerve repair and decrease tissue inflammation (Guan et al., 2024). Specifically, M2-Exos reduce inflammation by facilitating the transformation of M1 pro-inflammatory macrophages into M2 anti-inflammatory macrophages, which establishes a microenvironment supportive of tissue repair (Guan et al., 2024). Furthermore, the controlled release of exosomes from hydrogels ensures that they remain at the injury site for extended periods, addressing the limitations of direct injection methods that often result in rapid exosome elimination (Kalluri and McAndrews, 2023). This approach is further enhanced by combining NTFs with hydrogel scaffolds, which work synergistically to improve neural tissue repair and restore functional capabilities (Bajic et al., 2024).

The application of exosome-integrated hydrogels extends beyond neural repair to other tissue types, including bone and skin. Stem cell-derived exosomes show promise for bone regeneration by promoting both osteogenesis and angiogenesis, processes vital for bone healing (Lu et al., 2023). The use of GelMA hydrogels as an exosome delivery vehicle provides an optimal method for controlled release, thus facilitating the repair of critical bone defects (Lu et al., 2023). In cutaneous wound healing, the application of exosome-loaded hydrogels yields superior results by enhancing re-epithelialization, vascularization, and collagen deposition, ultimately leading to comprehensive skin restoration (Li et al., 2021). Furthermore, dual-sensitive hydrogels that detect pH and temperature shifts allow for more precise exosome delivery and release, maximizing therapeutic effectiveness at the wound site (Li et al., 2021).

In addition to exosome-based strategies, stem cell transplantation and NTF delivery represent important complementary approaches for clinical translation. In preclinical models, stem cell-derived motor neurons demonstrate potential for reconnecting with denervated muscles and restoring function (Tokutake et al., 2022). Furthermore, genetically modified stem cells engineered to overexpress BDNF may provide enhanced neuroprotection and promote axonal regeneration in denervated settings (Zimmermann et al., 2016). Optimizing the delivery timing and combination of NTFs, such as GDNF and IGF, alongside appropriate biomaterial carriers, improves cell survival and host integration (Tokutake et al., 2022). When combined with neurogenic hydrogel platforms, these strategies hold promise for advancing functional restoration in nerve-impaired wounds; however, their evaluation in validated animal models remains essential for future clinical translation.

## 7 Conclusion

Neuro-driven skin regeneration represents a multidisciplinary approach that combines neuroscience and regenerative medicine to address complex challenges in tissue repair. This strategy depends on the natural synergy between nervous system functions and skin healing processes, and shows promise for treating denervated wounds, such as diabetic foot ulcers and pressure sores. Because the skin maintains a functional connection with the peripheral nervous system, neural signals can perform sensory functions while simultaneously supporting the repair of epidermal and dermal tissues. Because denervation creates a physiological imbalance that results in poor wound healing, neuro-driven approaches are necessary to restore this complex regulatory system.

These strategies function as a sophisticated treatment framework for both acute skin defects and chronic wounds. The regenerative capabilities of skin-derived precursors are being leveraged to advance the development of skin substitutes, 3D bioprinting techniques, extracellular vesicles, and NTF delivery systems. The advancement of these technologies provides opportunities for enhancing the quality of life for patients with complex skin injuries and will continue to drive progress in the field of regenerative medicine.
